# CPT to RVU conversion improves model performance in the prediction of surgical case length

**DOI:** 10.1038/s41598-021-93573-2

**Published:** 2021-07-08

**Authors:** Nicholas Garside, Hamed Zaribafzadeh, Ricardo Henao, Royce Chung, Daniel Buckland

**Affiliations:** 1 Department of Mechanical Engineering and Materials Science, Duke University, Durham, NC USA; 2 Department of Surgery, Duke University School of Medicine, Durham, NC USA; 3 Department of Biostatistics and Bioinformatics, Duke University School of Medicine, Durham, NC USA

**Keywords:** Computational biology and bioinformatics, Health care, Medical research, Engineering

## Abstract

Methods used to predict surgical case time often rely upon the current procedural terminology (CPT) code as a nominal variable to train machine-learned models, however this limits the ability of the model to incorporate new procedures and adds complexity as the number of unique procedures increases. The relative value unit (RVU, a consensus-derived billing indicator) can serve as a proxy for procedure workload and could replace the CPT code as a primary feature for models that predict surgical case length. Using 11,696 surgical cases from Duke University Health System electronic health records data, we compared boosted decision tree models that predict individual case length, changing the method by which the model coded procedure type; CPT, RVU, and CPT–RVU combined. Performance of each model was assessed by inference time, MAE, and RMSE compared to the actual case length on a test set. Models were compared to each other and to the manual scheduler method that currently exists. RMSE for the RVU model (60.8 min) was similar to the CPT model (61.9 min), both of which were lower than scheduler (90.2 min). 65.2% of our RVU model’s predictions (compared to 43.2% from the current human scheduler method) fell within 20% of actual case time. Using RVUs reduced model prediction time by ninefold and reduced the number of training features from 485 to 44. Replacing pre-operative CPT codes with RVUs maintains model performance while decreasing overall model complexity in the prediction of surgical case length.

## Introduction

Hospital systems within the United States commonly use the current procedural terminology (CPT) coding system developed by the American Medical Association (AMA) to identify specific procedures performed during surgery^1,2^. Nationally implemented in the 1980s, CPT codes are standardized by the AMA to streamline hospital operations and billing processes. As of 2020, there exist thousands of unique 5-digit CPT codes ranging from 00100–99499, grouped by specialty and type. The surgical category ranges from 10021–69990. Anticipated procedures are logged by hospital operations in the form of pre-operative (pre-op) CPT codes, which may differ from procedures that end up being performed (the post-op CPT list) based on clinical findings during the operation.

These nominal CPT codes have been used extensively in the development of empirical models that predict operating room (OR) surgical case length. Wright et al*.*^3^, for example, showed that regression models utilizing surgeon estimates and mapped categorical CPT codes could perform as well as scheduling system estimations, with a mean absolute error (MAE) of 55 min. Eijkemans et al*.*^4^ used 253 unique procedure categories to develop their models, which attained a 25% reduction in MAE compared to that of human-estimated scheduling predictions. More complex machine-learned (ML) models have been developed since these early regression models^5–8^, but all have relied on categorical CPTs in their predictions, which limits applicability beyond their specific training scope. Furthermore, high complexity in these ML algorithms, such as the gradient-boosted decision tree developed by Zhao et al*.* in 2019, can face longer training times and higher computational costs compared to their simpler regression predecessors.

In 1991, the AMA began reimbursing hospitals based on the relative value unit (RVU) which is a standardized index associated with each CPT that captures staff workload for a given procedure^9^. The total RVU is a combination of three separate measures: work RVU, practice expense RVU, and professional liability RVU^10^. In most cases, work RVU is the dominant term in the total RVU sum, while the other two terms are sufficiently small to be ignored. For sense of scale, typical RVU values fall in range [0, 100]. Like other indices meant to capture relative intensity of a single characteristic^11^, the RVU places a lower-bound at zero and increases indefinitely. It may also provide added benefits in model simplicity, much like other clinical indices in recent work^12^. We hypothesize that conversion of CPT codes into their respective work RVU indices will serve as supplement for capturing case complexity in the prediction of OR case length, maintaining model performance while increasing the model’s ability to perform outside a subset of specifically trained surgical procedures.

## Methods

### Cohort identification and data preprocessing

Retrospective billing data of non-emergent colorectal and spinal surgeries from Duke University Health Systems (with 6 separate locations) between July 2014 and December 2019 was collected and deidentified. This work was found exempt by the Duke Institutional Review Board (Pro00104275). After removing records with missing values and/or errors in the timestamps, we obtained 11,696 inpatient/outpatient records for analysis, including 3847 colorectal and 7849 spinal surgery cases with average case time of 218 min (SD: 139 min) for the entire cohort. This includes 27 patients under the age of 18 (min age, 13). For the colorectal subspecialty, the average case time was 180 min (SD: 156 min). For the spinal subspecialty, the average case time was 236 min (SD: 126 min). The total time of surgery was calculated in minutes from operating room setup to cleanup time and then log-transformed to handle skewness. A best-subset regression process was followed to choose a combination of predictor variables from the retrospective billing data that could be correlated with case length as the target output of a linear regression model. This exploratory data analysis revealed nine significantly correlated pre-op features that maximized the adjusted R^2^ of the tested linear regression models. These variables included: CPT code, work RVU, number of panels (surgical specialties involved), number of procedures, geographic location (buildings within Duke Health System), patient class, first case of the day (binary), day of the week, and primary physician. The nominal CPT code variable comprises a list of the top 10 CPT codes used to classify the scheduled case, which were also used to derive a summed work RVU index based on Center for Medicare and Medicaid Services (CMS) Physician Fee Schedule January 2020 released file^13^. Importantly, this is the physician work RVU, but will be referred to as “RVU” through the remainder of this study. For the colorectal subspecialty, the average RVU was 16.7 (SD: 19.6). For the spinal subspecialty, the average RVU was 42.4 (SD: 23.0). For the entire cohort, the average RVU was 33.9. All variable descriptions and high-level distributions are listed in Tables 1, 2 and 3.

**Table 1:** Feature descriptions.

Variable	Type	Description
CPT code	Nominal	Current procedural terminology—a five-digit code representing procedure ID
RVU	Continuous	Relative value unit—measure of procedure complexity for use in billing
Number of panels	Ordinal	Number of surgical specialties needed for each case
Number of procedures	Ordinal	Number of procedures performed in each case
Location	Nominal	One of six buildings within Duke University Health System, both outpatient and inpatient
Patient class	Nominal	Patient class labels as ambulatory surgery, surgery admit inpatient, inpatient, surgery bedded outpatient, emergency
First case flag	Nominal	A binary flag indicating the first case of a day for a given operating room
Day of the week	Nominal	Day that the operation was performed (Mon, Tue, Wed, Thu, Fri, Sat, Sun)
Primary physician ID	Nominal	Identifier tied to lead surgeon on case

**Table 2:** Continuous variable distributions.

Variable	25% Percentile	Mean	Median	75% Percentile
RVU	13.2	34.0	33.3	48.1
Number of panels	1	1.1	1	1
Number of procedures	1	4.4	4	7

**Table 3:** Categorical variable distributions.

Variable	Unique count	Most common value percentage
CPT	443	17.1
Location	6	45.4
Patient class	5	42.7
First case flag	2	65.2
Day of the week	7	26.3
Primary physician	26	10.5

### Model development and evaluation

Three extreme gradient boosting (XGBoost, version 1.1.1)^14^ models were developed in Python (version 3.7.6) to predict total time of the surgery. These models utilized either the RVU, the CPT codes, or the RVU and the CPT codes combined while the remaining features remained the same in each model. The models were trained to minimize the mean squared error (MSE) as its loss function. Training and hyper-parameters optimization were performed using a 75–25% training-test data split and by fivefold cross validation, in which different combination of hyper-parameters were evaluated in each of the five randomized training subsets. The best-performing hyper-parameters were selected to minimize the average mean squared error across the five subsets. These hyper-parameters include maximum tree depth, subsample ratio, and L1- and L2-regularization terms. Then, the model with the best hyper-parameters was used to predict the length of surgery within the test data (i.e. 25% of the data) and finally evaluated using mean absolute error (MAE) and root mean squared error (RMSE) as the performance metrics. To calculate the 95% confidence interval of the performance metrics, we used bootstrapping and resampling methods on the test data set.

## Results

### Schedulers performance

**Figure 1:**
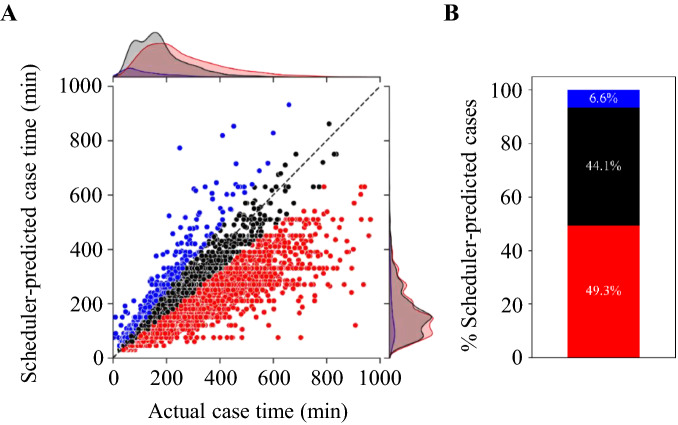
Overview of schedulers performance. (**A**) Distribution of the actual and the scheduler-predicted case time in minutes, (**B**) percentage of cases predicted under, over, and within 20% of the actual case time. Scheduler-predicted case time within 20%, and > 20% under or over the actual case time are depicted in black, red, and blue, respectively.

To provide a better perspective on scheduler performance, we first defined 20% of the actual length of surgery as the acceptable margin of error, meaning any prediction below or above this threshold was considered an under- or over-prediction, respectively. We then plotted all the data in a joint plot to visualize the distribution of each category. As shown in Fig. 1A, human schedulers under-predicted surgery time more often than over-predicted, with 49.3% of cases under-predicted, 44.1% on time, and 6.6% over-predicted (Fig. 1B).

### Model performance

**Figure 2:**
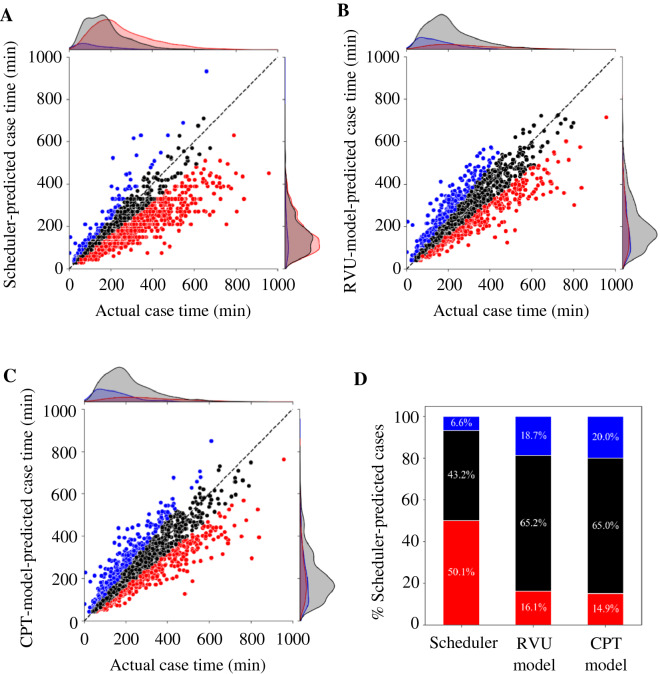
Overview of the models and the scheduler performance. (**A**) Distribution of the actual and the scheduler-predicted case time in minutes, (**B**) distribution of the actual and the RVU-model-predicted case time in minutes, (**C**) distribution of the actual and the CPT-model-predicted case time in minutes, (**D)** percentage of cases predicted under, over, and on-time. Predicted case time within 20%, and > 20% under or over the actual case time are depicted in black, red, and blue, respectively.

The distributions of the model and the schedulers prediction using the test set are shown in Fig. 2A–C. Figure 2D then reports the over- and under-prediction percentage distributions. They show that the model correctly predicted more cases within the 20% margin of error comparing with the scheduler, with less under-prediction and more over-prediction. The model predicted 65.2% of cases within the 20% margin of error (51% improvement over the scheduler), under-predicted 16.1% of cases (67.9% improvement over the schedulers), but over-predicted 18.7% of cases (283.3% increase over the scheduler, Fig. 2C). The MAE and RMSE values of the schedulers, which were calculated from the test set, were 60.1 and 90.2 min, respectively. The model which uses only the RVU, outperformed the schedulers performance by 35.6% and 32.6% in terms of MAE and RMSE and reduced them to 38.5 and 60.8 min, respectively (Table 4). Similarly, the model which used the CPT codes instead of the RVU also improved the schedulers MAE and RMSE by 34.4% and 31.4%, respectively but required 9.4-times more time than the RVU model to predict the case length. On the other hand, the model that uses both the RVU and the CPT codes improved the RVU model MAE and RMSE only by 2.3% and 2.2%, respectively and required 8.5-times more time to predict the case length.

**Table 4:** Model and scheduler performance metrics.

Prediction type	Prediction time (relative to RVU model)	MAE (95% CI)	RMSE (95% CI)
Scheduler	N/A	60.1 (57.0–63.6)	90.2 (84.7–96.5)
RVU model	1	38.5 (36.3–40.9)	60.8 (56.3–66.0)
CPT model	9.4	39.4 (37.0–41.9)	61.9 (56.3–67.4)
RVU + CPT model	8.5	37.3 (34.9–39.6)	58.8 (54.0–63.8)

### Feature importance

**Figure 3:**
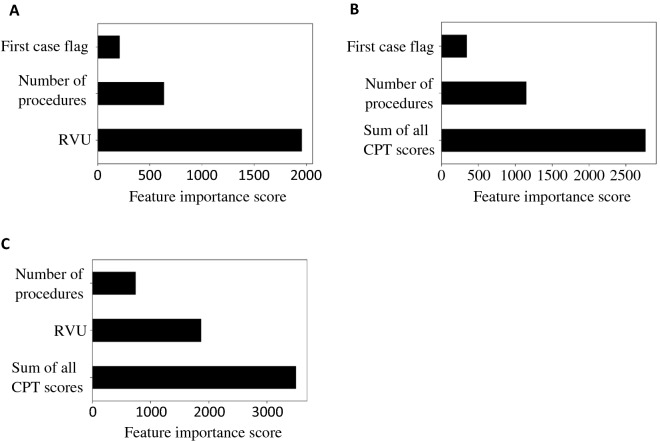
Features importance comparison. The average number of times each feature was utilized to split the data across all the trees in the model with (**A**) RVU-based, (**B**) CPT-based, and (**C**) RVU/CPT combined.

To compare the importance of features in construction of the boosted trees, we calculated the average number of times each feature was utilized to split the data across all the trees as the “score” of each feature. Figure 3 shows the top 3 features that were most used in each model. RVU (score = 1960) and all the CPT codes (score = 2773) were the most important features in the RVU and CPT models, respectively (Fig. 3A,B). Interestingly, the RVU (score = 1880) was the second most important feature after the all the CPT codes (score = 3509) in the model that incorporated the RVU and CPT codes (Fig. 3C).

## Discussion

The main goal of this study was to determine model performance effects from the CPT to RVU conversion. The RVU-based method not only succeeds in maintaining RMSE compared to the CPT-based method (Table 3) but yields a 1.1-min reduction, pointing to the possibility that RVU usage trends toward improved performance. While CPT and RVU were initially created to serve different functions by design, these results show that both input types offer information on relative case complexity in the prediction of surgical case length. Preserving CPT codes as a nominal feature in model creation allows the algorithm to uncover case complexity implicitly, whereas conversion to RVU relies on a pre-determined magnitude of case complexity that has already been established by a board of experienced physicians. These comparisons clearly show that replacing the CPT codes with the RVU yields similar but more efficient performance in this specific cohort.

While performance between the CPT-based and RVU-based methods were similar, inference times (the time it takes to calculate a prediction) were not. Conversion to RVU showed a 9.4-fold reduction in total time to predict case time. This could be due to the reduced number of features (485 to 44), number of tree-based learners (70 to 60), and maximum depth (11 to 9) in RVU-based model. The reduced training time is important when the models use very large training datasets and need to be retrained frequently. This could also increase availability of computational resources for other models that would be running in parallel.

The RVU-based model shows increased robustness compared to its CPT-based counterpart. Besides sparsity, one disadvantage of using CPT’s in model creation is its dependence on familiar codes to be able to calculate an output. New CPT’s that were not part of the training data cannot be used. A CPT code from the cardiothoracic subspecialty, for example, would not generate a predicted surgical case time since no CPT’s were used in the training data. Other sources of model breakdown include data entry errors, non-existent CPT codes, or outdated procedure codes. RVU, on the other hand, is more robust as it is a continuous data type that avoids these sources of breakdown, enabling new and uncommon procedures to be handled with ease.

From Table 4 we see an improvement in MAE and RMSE measures for all three developed models compared to the human scheduler. In our data, human schedulers under-predict case length half of the time (Fig. 1B) and over 8 times as often as over-prediction, affecting the health systems ability to efficiently schedule room allocations and staffing costs. The improved accuracy and more balanced error performance of our predictive models could reduce associated room backlog and overtime cost problems if used on a consistent basis. Based on our results we would expect any of the three models to outperform the human scheduler in accuracy. However, it is important to acknowledge potential reasons for purposeful underprediction by the human scheduler. One benefit, for example, would be reduced room latency and increased number of procedures performed during the day. It remains to be seen if predictive model usage would lead to increased net revenue for the hospital, which would be a worthwhile direction for future work.

While conversion to RVU retains case complexity information for a given procedure, it is important to acknowledge that procedure identification information is lost in the process. This implies that two completely different procedures within different subspecialties will appear indistinguishable from one another if they have similar RVU values. While this may not be important for the problem of case length prediction, it may be undesirable when extending to other types of predictive models that use CPT codes to distinguish between procedures with similar work value. Another limitation of this study is that we didn’t explore every possible feature selection method (best subset regression), and future methods may use a different feature subset in training. This study only looks at two surgical subspecialties: colorectal and spine. Although this cohort covers broad ranges of case time (mean: 218, SD: 139) and RVU (mean: 34, SD: 25), RVU-based model performance might change with data inclusion from other subspecialties and with more observations from the colorectal and spinal subspecialties. Moreover, this study used data from multiple locations within a single, large hospital system which uses the same scheduling methods. Therefore, future studies could show how the RVU-based model would perform in other locations and surgical specialties beyond its limited, non-emergent training scope.

## Conclusion

Converting the categorical CPT code to an RVU that captures case complexity provided similar model performance in predicting colorectal and spinal surgical case length. The conversion enabled shorter model training and prediction times and increases robustness to unanticipated procedure inputs.

## Data Availability

The data used in this study is considered privileged under HIPAA within Duke Health System due to many patient specific identifiers used in the model training and is unavailable for public release. A de-identified data set may be provided on request after institutional approval.

## References

[1] Thorwarth W (2004). From concept to CPT code to compensation: How the payment system works. J. Am. Coll. Radiol..

[2] CPT^®^ overview and code approval. (n.d.). Retrieved December 02, 2020, from https://www.ama-assn.org/practice-management/cpt/cpt-overview-and-code-approval

[3] Wright I, Kooperberg C, Bonar B, Bashein G (1996). Statistical modeling to predict elective surgery time: Comparison with a computer scheduling system and surgeon-provided estimates. Anesthesiology.

[4] Eijkemans M, van Houdenhoven M, Nguyen T, Boersma E, Steyerberg EW, Kazemier G (2010). Predicting the unpredictable: A new prediction model for operating room times using individual characteristics and the surgeon's estimate. Anesthesiology.

[5] Strum DP, Vargas LG, May JH (1997). Surgical suite utilization and capacity planning: A minimal cost analysis model. J. Med. Syst..

[6] Zhao B, Waterman RS, Urman RD, Gabriel RA (2019). A machine learning approach to predicting case duration for robot-assisted surgery. J. Med. Syst..

[7] Bartek MA, Saxena RC, Solomon S, Fong CT, Behara LD, Venigandla R, Velagapudi K, Lang JD, Nair BG (2019). Improving operating room efficiency: Machine learning approach to predict case-time duration. J. Am. Coll. Surg..

[8] Stepaniak PS, Heij C, Mannaerts GH, de Quelerij M, de Vries G (2009). Modeling procedure and surgical times for current procedural terminology-anesthesia-surgeon combinations and evaluation in terms of case-duration prediction and operating room efficiency: A multicenter study. Anesth. Analg..

[9] Nurok M, Gewertz B (2019). Relative value units and the measurement of physician performance. J. Am. Med. Assoc..

[10] Coberly, S. (Ed.). (2015, January 12). The Basics: Relative Value Units (RVUs). Retrieved December 09, 2020, from https://www.nhpf.org/library/the-basics/Basics_RVUs_01-12-15.pdf

[11] Parr RG, von Szentpály L, Liu S (1999). Electrophilicity Index. J. Am. Chem. Soc..

[12] Glass RI, Mulvihill MN, Smith H, Peto R, Bucheister D, Stoll BJ (1977). The 4 Score: An index for predicting a patient's non-medical hospital days. Am. J. Public Health.

[13] RVU20A Physician Fee Schedule January 2020 Release. (2020, January 31). Retrieved March, 2020, from https://www.cms.gov/medicaremedicare-fee-service-paymentphysicianfeeschedpfs-relative-value-files/2020

[14] Chen, T., & Guestrin, C. XGBoost: A scalable tree boosting system. In *Proceedings of the 22nd ACM SIGKDD International Conference on Knowledge Discovery and Data Mining*, 785–794 (ACM, 2016). 10.1145/2939672.2939785

